# Interplay among Oxidative Stress, Autophagy, and the Endocannabinoid System in Neurodegenerative Diseases: Role of the Nrf2- p62/SQSTM1 Pathway and Nutraceutical Activation

**DOI:** 10.3390/cimb46070410

**Published:** 2024-07-02

**Authors:** Federica Armeli, Beatrice Mengoni, Debra L. Laskin, Rita Businaro

**Affiliations:** 1 Department of Medico-Surgical Sciences and Biotechnologies, Sapienza University of Rome, Corso della Repubblica, 79, 04100 Latina, Italy; federica.armeli@uniroma1.it (F.A.); beatrice.mengoni@uniroma1.it (B.M.); 2 Department of Pharmacology and Toxicology, Ernest Mario School of Pharmacy, Rutgers University, Piscataway, NJ 08854, USA; laskin@eohsi.rutgers.edu

**Keywords:** Nrf2, p62/SQSTM1, oxidative stress, autophagy, endocannabinoid system, nutraceuticals, neuroinflammation, neurodegenerative diseases, neuroprotection

## Abstract

The onset of neurodegenerative diseases involves a complex interplay of pathological mechanisms, including protein aggregation, oxidative stress, and impaired autophagy. This review focuses on the intricate connection between oxidative stress and autophagy in neurodegenerative disorders, highlighting autophagy as pivotal in disease pathogenesis. Reactive oxygen species (ROS) play dual roles in cellular homeostasis and autophagy regulation, with disruptions of redox signaling contributing to neurodegeneration. The activation of the Nrf2 pathway represents a critical antioxidant mechanism, while autophagy maintains cellular homeostasis by degrading altered cell components. The interaction among p62/SQSTM1, Nrf2, and Keap1 forms a regulatory pathway essential for cellular stress response, whose dysregulation leads to impaired autophagy and aggregate accumulation. Targeting the Nrf2-p62/SQSTM1 pathway holds promise for therapeutic intervention, mitigating oxidative stress and preserving cellular functions. Additionally, this review explores the potential synergy between the endocannabinoid system and Nrf2 signaling for neuroprotection. Further research is needed to elucidate the involved molecular mechanisms and develop effective therapeutic strategies against neurodegeneration.

## 1. Introduction

The common hallmarks of neurodegenerative diseases, such as Alzheimer’s disease (AD) and Parkinson’s disease (PD), include the accumulation of protein aggregates, oxidative stress, altered autophagy, and chronic neuroinflammation leading to neuronal death. Treatments aimed at targeting these fundamental processes could have broad clinical applicability. So far, several theories have been proposed to relate these processes to neurodegenerative diseases. However, it is very difficult to find a correlation among all the multiple factors that have been hypothesized upstream to the development of neurodegenerative diseases [1]. For example, it is known that the increase in protein aggregates, typical of neurodegenerative diseases, leads to increased oxidative stress [2,3]. In this context, this review aims to investigate the correlation between oxidative stress and autophagy in neurodegenerative diseases. Autophagy is one of the main cellular pathways associated with neurodegenerative diseases, and there is an important reciprocal interaction between autophagy and oxidative stress that could lead to the development of new therapeutic strategies. Reactive oxygen species (ROS) are byproducts of normal metabolism and play important roles in many biological processes including cellular homeostasis, signaling, and autophagy. Several factors, such as aging, and also genetic factors, can compromise the normal function of redox signaling with increased ROS formation and oxidative stress [1]. Several studies claim that climate change has multiple effects on living organisms. When the outside temperature varies by even one degree because of intensified global warming, a process of acclimatization begins that causes, if protracted, the activation of biochemical pathways ultimately connected to neurodegeneration, such as oxidative stress, excitotoxicity, and neuroinflammation. For instance, heat stress can induce protein misfolding resulting in increased protein aggregates. In animal models, hyperthermia causes an AD-like molecular phenotype with upregulation of Aβ expression and deposition of phosphorylated tau [4]. At the brain level, redox signaling is involved in fundamental functions, such as neuronal differentiation, plasticity, and memory consolidation [5]. Neurons, because of their high oxygen uptake and low levels of antioxidants, easily suffer oxidation, and considering that they are post-mitotic cells that accumulate a large amount of oxidized molecules, it is known that large amounts of ROS lead to neuronal death [6]. Indeed, increased oxidant factors as well as a deficiency of antioxidant enzymes are common markers of neurodegenerative diseases [7]. The activation of nuclear factor erythroid-related factor 2 (Nrf2) is among the main antioxidant mechanisms. Nrf2 is a ubiquitous transcription factor activated by oxidative stress that binds the antioxidant response element (ARE), an enhancer sequence present in the regulatory regions of antioxidant genes [8]. Nrf2 is expressed in both glial cells and neurons [9]. In AD, Nrf2 expression is highest in the cytoplasm of hippocampal neurons, while under physiological conditions, its expression is highest in the nucleus, where it induces an antioxidant response [10]. As a matter of fact, Nrf2 knockout mice show increased susceptibility to neurodegeneration [11]. Autophagy is a cellular process involved in maintaining cellular homeostasis; it deals with the degradation and recycling of damaged intracellular organelles and misfolded proteins [12]. The role of p62/sequestosome-1 (p62/SQSTM1) on tau protein metabolism and neurofibrillary tangle (NFT) formation is well known, and the regulation of autophagy flux has also been seen to improve Alzheimer’s symptoms [13,14]. The presence of NTFs is an important histopathological lesion in AD. A new constituent of neuropathological protein aggregates, the protein p62/SQSTM1, binds ubiquitinated proteins, which suggests that the accumulation of ubiquitin-conjugated proteins and p62/SQSTM1 in cytoplasmic inclusions might be related. NFTs show immunoreactivity for ubiquitin-binding protein p62/SQSTM1, and this immunoreactivity appears early during neurofibrillary pathogenesis and is stably present in NFTs. This suggests that dysfunction in ubiquitin-mediated proteolysis and the subsequent accumulation of ubiquitin-conjugated proteins may contribute to the origin of NFTs. This indicates that the early involvement of p62/SQSTM1 might be critical in the formation of NFTs [15]. The involvement of p62/SQSTM1 in relation to Aβ deposition in AD has not been elucidated yet, although increased expression of p62/SQSTM1 leads to reduced deposition of Aβ42/Aβ40 in the hippocampus of AD animal models [16]. 

## 2. Triggering of the p62/SQSTM1-Keap1-NRF2 Pathway

**Figure 1:**
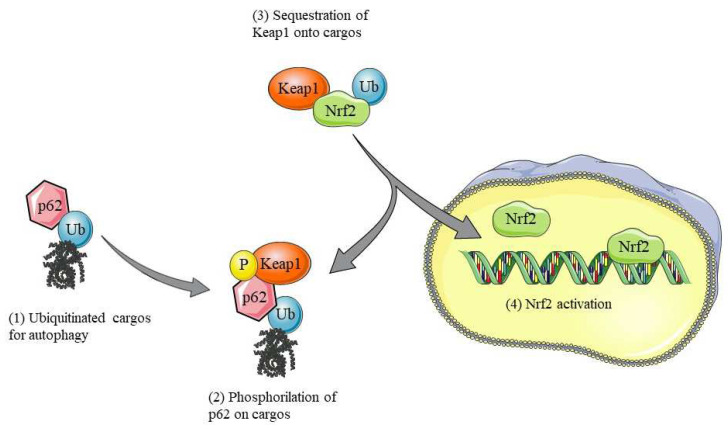
Interaction among p62/SQSTM1, Nrf2, and Keap1. The p62/SQSTM1 protein acts as a key bridge between Keap1 and Nrf2, facilitating the release of Nrf2 from the Keap1 complex and thus allowing its translocation into the nucleus. Once in the nucleus, Nrf2 activates the transcription of genes involved in cellular defense against oxidative stress and the maintenance of homeostasis. This mechanism is crucial for the cellular response to external stimuli and for maintaining cellular health.

Nrf2 is the main factor involved in the regulation of antioxidant enzyme expression in response to oxidative stress. In physiological conditions, Nrf2 is sequestered in the cytoplasm by Keap1 (Kelch-like ECH-associated protein 1), which transports it to the proteasome for its degradation. Although Keap1 molecules bind to Nrf2 at a 2:1 ratio, a fraction of Nrf2 escapes the Keap1 complex by translocating into the nucleus, allowing basal gene expression of antioxidant genes. Indeed, one study suggests that in conditions of cellular homeostasis, as a result of the balance between the rate of Nrf2 synthesis and degradation, a constant level of Nrf2 protein is nevertheless maintained [17]. Mass spectrometry studies, using the 21-mesylated dexamethasone electrophile, provide evidence that Keap1 contains stress sensors [18]. Through these studies and by exploiting point mutations, cysteine residues of Keap1 required for its function have been identified. In addition, some of the Nrf2-activating compounds are able to oxidize cysteine residues Cys151, Cys273, and Cys288, critical in the Keap1-mediated stress response [19]. In the presence of oxidative stress, therefore, the oxidation of Keap1 cysteines allows for the dissociation of the Nrf2-Keap1 complex, resulting in the translocation into the nucleus of Nrf2 [20]. Within the nucleus, Nrf2 interacts with Maf transcription factors by binding to the cis-regulatory sequences of ARE [21]. This provides coordinated expression of antioxidant enzymes and cytoprotective proteins that enhance the elimination of ROS. p62/SQSTM1 has emerged as a versatile adaptor protein able to play various biological functions through its interaction with numerous other proteins. In particular, p62/SQSTM1 plays a key role in autophagy, functioning as a transporter of substrates to the proteasome for their degradation [22]. In addition, p62/SQSTM1 directly interacts with Keap1 and disrupts the association between Keap1 and Nrf2, leading to Nrf2 stabilization and nuclear accumulation (Figure 1). In contrast, the p62/SQSTM1 gene is a target of Nrf2, so Nrf2 stimulates p62/SQSTM1 production. The combination of these two branches forms a positive feedback loop in the antioxidant response (Nrf2 induces p62/SQSTM1 and p62/SQSTM1 induces Nrf2). It has also been reported that p62/SQSTM1 determines the expression of Keap1 at the basal level and its activity. Normally, after the completion of autophagy, the increase in p62/SQSTM1 expression is balanced by an increase in its turnover. The recovery of Nrf2/Keap1 after oxidative insult occurs through the ability of p62/SQSTM1 to bind oxidized Keap1, leading to its degradation. Taguchi et al. demonstrated how the disruption of autophagy by the specific ablation of ATG7 in the liver leads to liver injury through increased constitutive activation of p62/SQSTM1 and Nrf2. Deletion of Nrf2 or p62/SQSTM1 in mice can ameliorate liver injury, while deletion of Keap1 exacerbates it [23]. Therefore, the inhibition of autophagy leads to negative consequences. It seems clear that Nrf2 and p62/SQSTM1 interact positively. Normally, p62-dependent autophagy of Keap1 would increase the turnover of oxidized Keap1, restoring the Nrf2/Keap1 system, and this seems to be a cellular defense mechanism under conditions of physiological stress. In this sense, the inhibition of autophagy leads to disruption of the Nrf2/Keap1/ p62/SQSTM1 system, which leads to reduced autophagic degradation of the p62/SQSTM1 complex and thus to the accumulation of cytopathic aggregates [24]. According to Salminen et al., in AD, the p62/SQSTM1 protein is associated with NFT composed mainly of hyperphosphorylated tau protein and ubiquitin. The p62/SQSTM1 protein is a multifunctional molecule with several domains that allow protein interactions. Through these interactions, p62/SQSTM1 participates in the regulation of cellular signals and the movement, aggregation, and degradation of proteins. p62/SQSTM1 can bind to ubiquitinated proteins by its UBA motif, controlling their aggregation and degradation through autophagy or proteasomes. It has been observed that p62/SQSTM1 is present in intracellular inclusions in neurodegenerative brain disorders with misfolded protein inclusions. Increasing evidence indicates that p62/SQSTM1 plays an important role in tau protein degradation. Studies have shown that the gene expression of p62/SQSTM1 and the cytoplasmic levels of p62/SQSTM1 protein are significantly reduced in the frontal cortex of AD patients. Decreased p62/SQSTM1 protein levels may disrupt Nrf2 signaling pathways, thereby increasing oxidative stress and impairing neuronal survival [25]. In fact, the study by Joshi et al. showed that the ablation of Nrf2 increases amyloid deposition and neuroinflammation and increases intraneural vesicles in the APP (Swe)/PS1ΔE9 mouse model of AD [26].

## 3. Autophagy: Molecular Mechanisms

Autophagy is a critical degradation process that involves the disassembly and recycling of cytosolic elements such as damaged organelles and misfolded proteins, as well as the clearance of pathogens [27]. Autophagic processes are extensively documented in mammalian systems, but several of the mechanistic determinations on the functioning of autophagy and the regulation of its molecular pathways have been made in yeast (*Saccharomyces cerevisiae*) [28]. Three kinds of autophagy exist including macro-autophagy, micro-autophagy, and chaperone-mediated autophagy. Macroautophagy carries the cargo for degradation to the lysosome through the intermediary of an autophagosome, which merges with the lysosome to form the autolysosome. In microautophagy, cellular elements are taken up through the invagination of the lysosomal membrane. In chaperone-mediated autophagy, instead, the proteins to be degraded bind to a chaperone protein complex that enables their translocation into the lysosomal membrane, resulting in unfolding and degradation [29]. Macroautophagy is the most studied process; it is stimulated by cellular stressors such as cellular nutrient deficiency, protein accumulation, and inflammation [29]. Hence, in this review, we will focus on the molecular aspects of macroautophagy (“autophagy”) and how it is also regulated under pathological conditions. 

**Figure 2:**
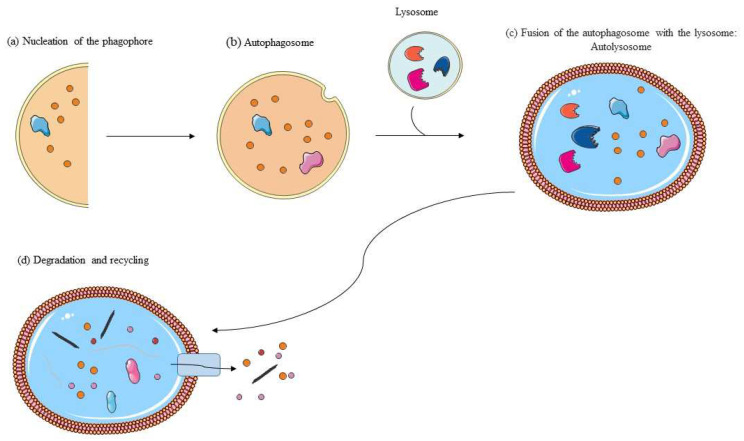
Autophagy is a complex self-degradation process that involves the following key steps: (**a**) phagophore formation; (**b**) maturation of the autophagosome; (**c**) fusion with the lysosome; and (**d**) degradation by lysosomal proteases of engulfed molecules and recycling.

Autophagy takes place through three key steps including the following (Figure 2): (a) nucleation of the phagophore; (b) formation and maturation of the autophagosome with the ingestion of selective cytoplasmic material to be degraded; (c) fusion of the autophagosome with the lysosome; and (d) proteolytic degradation of the engulfed material and recycling of the components [29].

The initiation of the autophagic process is inhibited by the kinase mTOR (mammalian target of rapamycin). mTOR is responsible for the formation of mTOR complex 1 (mTORC1), which inhibits catabolic pathways such as autophagy. mTORC1 is a downstream PI3K/AKT signaling pathway, and its activity is controlled by a signaling network involving Ras/Raf/MEK/ERK. In physiological conditions, nutrient deprivation inhibits mTORC1 activity and induces autophagy. The initiation of autophagy by ULK1 is reciprocally regulated by mTORC1 and AMPK: AMPK activates ULK1; when nutrients are sufficient, mTOR prevents the interaction between ULK1 and AMPK and thus the activation of ULK1. Autophagy initiation is also mediated by the VPS34 complex where Beclin-1 plays a key role: AMPK, through the phosphorylation of Beclin-1, promotes its dissociation from Bcl-2, allowing the autophagy process to start [30]. After autophagy onset, ATG7 recruits all other proteins related to autophagy and the formation of the phagophore begins, which then enlarges to become an autophagosome [31,32]. 

Autophagosome formation is completed by MAP1LC3/LC3 (ATG8 in yeast), which is subjected to proteolytic cleavage by ATG4, generating LC3-I. The C-terminal glycine residue of LC3-I binds the phosphatidylethanolamine (PE), leading to the formation of LC3-II [33]. LC3-II binds to the autophagosome membrane. Autophagosomes are later fused with the lysosomal membrane to make autolysosomes. Beclin-1 is decreased in the brains of mouse models and in AD patients, with a greater accumulation of immature autophagosomes and Aβ [34]. In mouse models of AD, mTOR inhibition reduces Aβ peptide levels and improves cognitive performance [35,36]. Cell components for autophagosome are “marked” with ubiquitin, which is identified by selective receptors, such as p62/SQSTM1, that “confiscate” and carry them to the autolysosome [37]. 

Autophagy is implicated in aging and neurodegeneration. The lack of homeostasis between free radical generation and repair mechanisms is responsible for aging, leading to oxidative stress [38]. Autophagy has been shown to be strongly correlated with oxidative stress through the direct interaction between p62/SQSTM1 and Keap1 [39]. Nrf2 and its inhibitor, Keap1, constitute an evolutionarily conserved cellular defense system to counteract oxidative stress. Normally, Nrf2 is retained by Keap1 in the cytoplasm, but in the presence of oxidative stress, it separates from Keap1 and migrates into the nucleus. Once there, it forms a complex that recognizes sequences ARE, which are crucial for activating the genes responsible for the antioxidant response, recruiting the factors necessary for the initiation of transcription. During the aging process in humans, there may be an alteration in the communication among Nrf2, Keap1, and p62/SQSTM. Indeed, Nrf2 knockout mice are more vulnerable to liver and lung diseases, neurodegeneration, and inflammatory stress [40,41,42]. The phosphorylation of the p62/SQSTM1 protein increases its binding affinity to Keap1, thus disrupting its binding with Nrf2. Consequently, the phosphorylation of p62/SQSTM1 promotes the expression of Nrf2 protective target genes. p62/SQSTM binds ubiquitinated protein aggregates and transports them to autophagosomes. The Keap1-Nrf2 pathway and autophagy are interconnected through the phosphorylation of p62/SQSTM1. In normal cells, this functional interaction serves as a defense mechanism, leading to the expression of antioxidant enzymes and the degradation of cytotoxic structures. When there are autophagic loads such as ubiquitinated protein aggregates and damaged mitochondria, the S403 residue in the ubiquitin-associated domain of p62/SQSTM1 is phosphorylated in a mTORC1-dependent manner, increasing the affinity of p62/SQSTM1 for Keap1. Consequently, Nrf2 translocates into the nucleus. The ubiquitinated loads, along with phosphorylated p62/SQSTM1 and the Keap1 complex, are degraded through autophagy, eliminating cytotoxic components. The activation of mTORC1 stimulates the expression of proteins that interact with the p62/SQSTM1/Keap1 complex. This complex is then degraded by autophagy, leading to the activation of Nrf2 [41]. It has been shown that sestrins protect cells from oxidative stress by inducing the degradation of Keap1 and upregulating Nrf2 activity, with this degradation mediated by autophagy. Additionally, the sestrin-induced degradation of Keap1 does not occur in the absence of p62/SQSTM1. p62/SQSTM has been identified as a protein that activates Nrf2 by disrupting the Keap1-Nrf2 interaction in cells with compromised autophagy, leading to the accumulation of p62/SQSTM1. The activation of Nrf2 by sestrins, therefore, facilitates the degradation of Keap1 by promoting p62-dependent autophagy, thus protecting cells from oxidative damage [43]. The p62/SQSTM1 and Nrf2 signaling pathways are strongly involved in cell survival by protecting against neurodegeneration [44]. Considering that ubiquitin plays a crucial role in the elimination of misfolded proteins, such as α-synuclein in PD and amyloid plaques in AD and damaged organelles, p62/SQSTM1 represents a suitable target for the modulation of proteasomal pathways [45]. Research conducted on mice lacking the p62/SQSTM1 protein has unequivocally demonstrated that the absence of this protein leads to neuropathological lesions, including the accumulation of hyperphosphorylated tau and NFT [46]. The protein p62/SQSTM1 is essential for transporting damaged mitochondria to autophagic degradation [25]. Mitochondrial instability is considered one of the early events in the onset of AD. Studies conducted in vitro on transgenic mice and on the post-mortem brains of AD patients have demonstrated that the accumulation of C-terminal fragments of the amyloid precursor protein leads to defects in the selective removal of mitochondria (mitophagy), increasing the production of ROS and compromising the basic degradation of mitochondria, as indicated by the accumulation of p62/SQSTM1 [47]. 

The protein p62/SQSTM1 undergoes degradation through autophagy. The lack or insufficiency of autophagy leads to an accumulation of p62/SQSTM1, thereby triggering persistent activation of Nrf2. Conversely, Nrf2 stimulates the expression of p62/SQSTM1, creating a positive feedback loop between Nrf2 activation and p62/SQSTM1 expression. p62/SQSTM1 acts as a bridge, connecting selective autophagy and the ubiquitination system to the oxidative stress response and redox regulation. Maintaining the homeostasis of p62/SQSTM1 levels is crucial for neuronal health [46]. Kanninen et al. highlighted that high expression of Nrf2 could ameliorate the symptoms of AD in transgenic AD mice [48]. Low nutrient levels activate autophagy to restore homeostasis through the degradation of macromolecules to provide nutrients [49]. Recently, it has been shown that these two cellular pathways directly intersect at the level of p62/SQSTM1, which, through interaction with Keap1, promotes the translocation of Nrf2 into the nucleus. The p62/SQSTM1-Nrf2 pathway is involved in autophagy and the anti-oxidative stress response; dysregulation of these pathways is associated with human disease–pathogenic mechanisms [41].

## 4. p62/SQSTM1-Nrf2 Pathway: A Target in Neurodegenerative Disease Therapeutic Approaches

Neuronal mitochondrial dysfunction and oxidative stress are the common features of several neurodegenerative diseases such as AD and PD, which result in excess ROS production [50,51]. These failures compromise oxidative phosphorylation, internal membrane integrity, and a malfunction in Ca^2+^ metabolism, triggering a cascade of processes leading to neuronal death [44].

Since no pharmacological therapies are available to reverse mitochondrial dysfunction, to date, the most successful approaches consist of antioxidant prevention. Plant-derived bioactive compounds and nutraceuticals are known to exert pleiotropic properties on inflammatory cells by reducing oxidative stress [52].

Polyphenols have well-documented anti-inflammatory effects; moreover, they increase the clearance of free radicals and ROS by modulating the activity of superoxide dismutase (SOD) and heme oxygenase-1 (HO-1) [53,54]. 

The powerful antioxidant activity of compounds obtained from the anthocyanin-rich blueberry has been extensively demonstrated. In vitro experimental trials have demonstrated the ability of blueberries to counteract LPS-induced inflammatory processes by inhibiting NF-kB signaling and oxidative stress [55,56]. These results have been confirmed by several in vivo studies in mice fed with hyperlipidic meals [57,58]. 

Furthermore, blueberry supplementation in the diet of obese adults or patients affected by metabolic syndrome has been shown to be beneficial by lowering inflammation and ROS in the blood, reducing oxidative stress, and decreasing indicators of type 2 diabetes mellitus such as methylamines, acetoacetate, acetone, and succinate in the urine. Real-time PCR analysis of the mRNAs of interleukin-6 (IL-6) and Transforming Growth Factor-β (TGF-β), pro- and anti-inflammatory cytokines, respectively, obtained from mononuclear blood cells, showed a significant decrease and increase. respectively, after blueberry supplementation, suggesting a reduction in inflammation [59,60]. 

In this scenario, antioxidant activity alone helps to counteract the inflammatory processes underlying neurodegenerative, cardiovascular, and metabolic diseases. The study of new therapeutic targets for the preventive treatment of neurodegenerative diseases is crucial. These targets would consider modulating the turnover of the mitochondrial pool in cells, like the elimination of damaged mitochondria (mitophagy), combined with antioxidant activity to preserve homeostasis in the brain [45,61]. In this connection, the focus of this review is on the Nrf2- p62/SQSTM1 regulatory pathway, which may potentially be an important target for drugs to fight neurodegenerative disorders.

In recent years, the Nrf2/ARE signaling pathway has become one of the most interesting targets for the therapy of these pathologies, not only as antioxidant protection. The maintenance of mitochondrial stability depends on a balance between mitochondrial biogenesis and mitophagy, processes that continually renew the mitochondrial pool. Signaling through the Nrf2/ARE pathway plays a crucial role in this mechanism, as it regulates the expression of genes involved in protection against oxidative stress, as well as in mitochondrial biogenesis and mitophagy. The protein p62/SQSTM1 is a multifunctional protein that acts as a selective receptor in mitophagy, facilitating the degradation of ubiquitinated substrates [44]. Mitophagy is a type of selective mitochondrial autophagy. The p62/SQSTM1 factor is made of several domains including the LIR domain that interacts with the C-terminal LC3, the ubiquitin-associated UBA domain, which binds autophagosomes to ubiquitinated proteins, and the KIR domain (Keap-interacting region) that binds Keap1 and induces nuclear translocation of Nrf2 [62].

In damaged mitochondria, the internal membrane is depolarized and PTEN-induced kinase 1 (PINK1) is degraded by proteases in the mitochondrial matrix, which accumulates on the outside membrane, leading to the activation of PARKIN, which is the cytosolic E3-ubiquitin ligase. PARKIN triggers mitophagy through protein ubiquitination and by recruiting the p62/SQSTM protein that interacts directly with the autophagosome [63]. The activation of the PINK1/PARKIN/p62/SQSTM1 axis plays an important role since it has been shown that the ablation of p62/SQSTM totally inhibits the clearance of damaged mitochondria [64]. Mitophagy is primarily orchestrated by PINK1, which is activated through self-phosphorylation and accumulates on the outer membrane of dysfunctional mitochondria. This accumulation of PINK1 activates the PARKIN E3 ubiquitin ligase, which in turn marks various proteins on the outer membrane of mitochondria with ubiquitin. This cascade signal facilitates the incorporation of damaged mitochondria into autophagosomes for their degradation. Because of abnormal interactions with Aβ in AD and α-synuclein in PD, mitophagic proteins like PINK1 and PARKIN are reduced, leading to alterations in mitophagy. Defective mitophagy, an emerging field of study, stems from compromised mitochondrial dynamics. It is essential to adopt a pharmacological approach urgently to enhance and/or restore mitophagy in neurodegenerative diseases [65]. There is evidence to suggest that Nrf2 may play a crucial role in mitochondrial biogenesis by actively participating in the elimination of damaged mitochondria through mitophagy. This seems to be particularly significant in situations of oxidative stress and mitochondrial damage [66]. In primary cultures of mouse hippocampal neurons, mutation of the amyloid precursor protein (APP) causes a reduction in the levels of proteins involved in the formation of mitochondria, such as Nrf2 and PINK1. This is associated with a defective antioxidant system regulated by Nrf2 and a reduced ability to eliminate damaged mitochondria, ultimately leading to neuronal degeneration [67]. Analogously to AD, mitochondrial integrity dysfunction is also linked to PD [66]. ROS production plays a significant role in maintaining the protein balance of α-synuclein, which is countered by the activity of Nrf2. Nrf2 reduces the formation of α-synuclein aggregates and protects against the loss of dopaminergic neurons. Indeed, the absence of Nrf2 intensifies the loss of dopaminergic neurons, neural inflammation, and protein aggregation [10,68]. In the murine model of PD induced by 6-hydroxydopamine (6-OHDA), a reduction in the protein expression of Nrf2 is observed [69]. The compromised antioxidant capacity and the deficit in mitochondrial clearance are elements involved in the mitochondrial dysfunctions observed in AD and PD. Consequently, it is plausible to suggest that a decrease in the expression or activity of Nrf2 may play a significant role in impairing mitochondrial biogenesis [66]. 

The ability of p62/SQSTM1 to up-regulate Nrf2 via Keap1 interaction was first described in 2010 [70,71,72]. In the same year, an ARE sequence was mapped in the promoter region of the gene encoding p62/SQSTM1, which is involved in its induction by oxidative stress via Nrf2 [73]. In 2019, p62/SQSTM1 knockdown was shown to reduce Nrf2 expression, associated with an increase in oxidative stress. Additionally, Nrf2 knockdown significantly reduces p62/SQSTM1 expression by negatively impairing autophagosome generation. These results support the positive feedback induced by p62/SQSTM1 in the Keap1-Nrf2 signaling pathway [74].

## 5. Endocannabinoid Activity and the Nrf2 Pathway

The neuroprotective powers of the Mediterranean diet have been largely demonstrated, and many studies have examined the nutritional balance of this diet as a factor modulating the endocannabinoid system (ECBS) [75]. Recent research has shown that the regulation of endocannabinoid (EC) tone may be a promising new therapeutic strategy to counteract neuroinflammation. Endocannabinoids perform many functions, influencing several processes that occur during both normal and pathological aging, including abnormal protein folding, inflammation, excitotoxicity, mitochondrial dysfunction, and stress, contributing to the maintenance of homeostasis by controlling several metabolic pathways [76]. The action of endocannabinoids (ECs) is mainly mediated through two different kinds of receptors including cannabinoid receptor 1 (CB1R) and cannabinoid receptor 2 (CB2R). CB1R receptors are predominantly located on nerve terminals and are abundant in the central nervous system (CNS). In contrast, CB2R receptors are present in cells of the innate immune system, such as microglia, and their activation regulates the expression of inflammatory mediators [75,77,78,79].

Increasing Nrf2/ECBS activity may be considered a promising therapeutic strategy to counteract neurodegenerative diseases [80]. Several studies have taken advantage of the cannabinoid pathway as an indirect way to promote Nrf2-mediated neuroprotection. In some studies, for example, primary cultures of neurons were exposed to Aβ oligomers and a high concentration of glucose. Subsequently, these cells were treated with several cannabinoid-related compounds, including endogenous or synthetic agonists. All of these compounds were shown to increase Nrf2 expression and protect the cells, improving their viability and concomitantly reducing Aβ levels and markers associated with inflammation and oxidative injury damage [81]. The activation of Nrf2 by the increased cannabinoid pathway appears to be an important feature, especially in microglia [82,83]. Microglia have CB2R receptors, and it is hypothesized that their activation controls the microglia response, thus preventing inflammatory damage in various models of neurodegenerative diseases [84,85].

Further evidence from studies on murine microglia cells, such as BV2, indicated that phytocannabinoids such as cannabidiol (CBD) and Δ9-tetrahydrocannabinol (Δ9-THC) are able to suppress proinflammatory NF-kB signaling pathways. Nrf2 indirectly inhibits NF-kB through HO-1 upregulation, which suppresses NF-κB activity itself [40,86]. The lack of Nrf2 could intensify NF-κB activity, causing increased cytokine production. The complex molecular mechanisms involving the Nrf2 pathway highlight the importance of developing more effective therapeutic strategies targeting Nrf2 to prevent or treat a wide range of neurological disorders [87]. CB2R receptor activation levels show a correlation with Aβ42 levels and plaque formation, indicating that such inflammatory processes could stimulate CB2R receptor expression. The significant increase in CB2R receptors in activated microglial cells may offer a therapeutic benefit by allowing Nrf2-targeted activation in damaged tissue areas [76,88]. Several investigations have highlighted the anti-inflammatory effects of CB2R receptor agonists in various models of AD. Accordingly, laboratory trials have shown that specific CB2R receptor agonists such as JWH-015, JWH-133, and HU-308 reduce the production of proinflammatory cytokines in microglial cell cultures exposed to Aβ peptide, inhibiting the transformation of microglia into a pro-inflammatory/neurotoxic M1 phenotype, and instead promoting their differentiation into an anti-inflammatory/neuroprotective M2 phenotype, an effect that has been observed to be reversed by Nrf2 inhibition [83,89,90,91]. These findings were corroborated by experiments in which specific CB2R receptor agonists were administered to mouse models after inoculation of Aβ in the brain. This resulted in a significant decrease in proinflammatory cytokines and reduced microglia reactivity [90,91,92]. A 2020 study on microglial cells showed that the CNR2 gene, which encodes for the CB2R receptor, hosts an ARE sequence in its promoter. This opens the possibility of using positive feedback between Nrf2 activation and upregulation of CB2R transcription [93].

The Mediterranean diet, through molecules such as β-caryophyllene (BCP), flavonoids, polyphenols, and vitamins, also enhances the activity of antioxidant systems through the activation of CB2R receptors [75]. A 2014 study revealed that Nrf2 translocation in the nucleus is partly influenced by CB2R receptor activation. In a C6 glioma cell model, increased ROS production, mitochondrial dysfunction, and oxidative stress were induced via glutamate exposure, causing cytotoxicity. Treatment with BCP induced the transfer of Nrf2 into the nucleus, restoring the antioxidant response and reducing ROS production. The phytocannabinoid compound BCP, a natural selective CB2R receptor agonist, activated Nrf2. Indeed, the CB2R antagonist AM630 neutralized the antioxidant effect of BCP in C6 glioma cells, suggesting that the antioxidant action of BCP is influenced by the expression of CB2R receptors [94]. Sirt1 also increases Nrf2 expression. Polyphenols such as neo-chlorogenic acid and resveratrol, found in various foods typical of the Mediterranean diet, are considered nutraceuticals capable of enhancing Sirt1 production. This, synergistically with Nrf2, increases the expression of PCG-1α, a crucial factor in counteracting neuronal mitochondrial dysfunction. This process promotes mitochondrial biogenesis, as well as antioxidant and anti-inflammatory activity to counteract neurodegeneration [95,96,97,98]. 

Growing evidence suggests that plant-based, anti-inflammatory diets, such as the Mediterranean diet, which is rich in fruits, vegetables, legumes, nuts, and whole grains, promote brain health. These diets contain bioactive compounds like antioxidant vitamins, polyphenols, other phytochemicals, and unsaturated fatty acids. Animal studies have demonstrated that these nutrients enhance neurogenesis and neuronal survival by mitigating oxidative stress and neuroinflammation [99].

## 6. Stimulation of the Nrf2 Regulatory Pathway as a Mechanism for Maintaining Homeostasis

Currently, there are still no compounds available for the regulation of p62/SQSTM1 expression. Further research is needed to develop them; however, several Nrf2 activators have been identified [44]. In addition to compounds currently undergoing clinical trials, there are other Nrf2 activators, such as curcumin, resveratrol, and the ketogenic diet, that represent promising candidates for the treatment of neurodegenerative disorders. Nutraceuticals demonstrate antioxidant and neuroprotective properties, providing promising insights for future clinical investigations. Specifically, the ethyl ester of ferulic acid, carnosic acid, sodium hydrosulphide, vanillic acid, sulforaphane, epigallocatechin-3-gallate, and resveratrol influence the Nrf2 transcription factor and have shown potential in slowing down the progression of AD in vivo [100,101]. It has emerged that a derivative of the phenylethyl ester of caffeic acid administered to mice with scopolamine-induced cognitive impairment protects against oxidative stress by facilitating the nuclear translocation and transcriptional activity of Nrf2 in both the hippocampus and cortex. This occurs by binding with Keap1 [102]. Epigallocatechin-3-gallate, a type of catechin found in high concentrations in green tea, has garnered attention for its potential to effectively stimulate Nrf2 [101]. In addition to increasing levels of SOD2, CAT, and GSH, epigallocatechin-3-gallate reduces the expression of pro-inflammatory cytokines and is capable of crossing the blood–brain barrier. This outcome may contribute to neuroprotective properties such as pro-autophagy and the suppression of misfolded protein aggregation [103,104,105,106]. There is evidence demonstrating that resveratrol also combats oxidative stress by increasing the activation of Nrf2 and facilitating its migration into the cellular nucleus. Additionally, it enhances the amount of mRNA encoding for Nrf2 [107]. Zhao et al. delved into the antioxidant mechanism of sulforaphane in an in vitro model of AD. Their findings revealed that sulforaphane increased the expression of Nrf2 and facilitated its movement into the cell nucleus by decreasing DNA demethylation levels at the Nrf2 promoter. Sesamol, the main component of sesame seed oil, has been highlighted for its anti-inflammatory and antioxidant effects [108]. In a 2018 study, the protective role of caffeic acid and resveratrol was explored in SK-N-SH cells transfected with the ataxin-3 gene and in ELAV-SCA3tr-Q78 transgenic Drosophila flies showing features similar to human spinocerebellar ataxia type 3. Caffeic acid and resveratrol treatment increased levels of antioxidant and autophagy protein expression with lower levels of reactive oxygen species through increased Nrf2 transcriptional activity that upregulates p62/SQSTM1 autophagy gene expression. Notably, blocking the Nrf2 pathway by siRNA reversed the effects of caffeic acid and resveratrol [109].

A study from 2018 proposed that administering sesamol could notably enhance the expression of heme oxygenase-1 and boost catalase (CAT) activity and glutathione (GSH) levels. This enhancement, in turn, could improve cognitive deficits induced by oxidative stress in mice with AD. Mechanistically, sesamol maintained a balanced cellular redox state, thus averting mitochondrial dysfunction and elevating antioxidant enzyme levels. This effect was achieved through the activation of the Nrf2 transcriptional pathway and its nuclear translocation [110]. In a rat model of PD triggered by 6-OHDA, the neuroprotective potential of ellagic acid was evidenced through its ability to heighten oxidative stress. This was emphasized by the elevated expression of monoamine oxidase B, Nrf2, and heme oxygenase-1, alongside a reduction in ROS levels within the striatum [111]. In another study in 2022, it was shown that treatment with thonningianin A, a polyphenolic compound found in natural plant-derived foods, significantly facilitated Nrf2 nuclear translocation by promoting p62/SQSTM1 interaction in zebrafish with 6-OHDA-induced ferroptosis and in dopaminergic neurons [112]. Ellagic acid was shown to counteract oxidative stress induced in the intrastriatal 6-hydroxydopamine rat model of Parkinson’s disease by improving Nrf2 concentrations [111]. 

Rats injected with Aβ into the CA1 region of the hippocampus and prefrontal cortex fed *Salvia macilenta* showed an increase in Nrf2 protein expression compared with the group injected with Aβ alone [113]. 

The role of nutraceuticals has been evaluated in several preclinical studies and a few clinical trials. A randomized, triple-blind, placebo-controlled trial showed that ellagic acid supplementation to Multiple Sclerosis patients was able to restore the Nrf2 decrease observed in these patients [114]. 

In Chinese AD patients taking curcumin for 6 months, a slowing of neuronal loss and improvement in cognitive function compared with the placebo group was detected [115]. Preclinical studies have demonstrated the neuroprotective effects of gypenosides in neurological disorders such as depression, PD, and AD by modulating important signaling pathways, including NF-κB, AKT, and ERK1/2, through activation of the Nrf2/KEAP1/ARE/HO-1 pathway [116]. Epigallocatechin-3-gallate has been shown to exhibit antioxidant, neuroprotective, and pro-autophagy properties by modulating the Nrf2/HO-1 antioxidant pathway [117]. Epigallocatechin-3-gallate-rich green tea has been associated with improved cognitive function in the elderly population [118]. However, it is important to note that the beneficial effects are carried out not by the single component but by the synergy of the combined components in green or black tea (epigallocatechin-3-gallate, theanine, or caffeine), thus showing a holistic effect [117]. Evidence from clinical studies on resveratrol supplementation has shown significant improvement in cognitive functions in healthy older adults and improvement in the integrity of the blood–brain barrier through an enhancement in the antioxidant system by activating the Nrf2 signaling pathway. Many studies confirm that resveratrol could positively modulate autophagy by reducing oxidative stress [117,119]. 

In recent years, there has been a surge in research on Nrf2, but the use of plant-based Nrf2 activators for neurodegenerative disorders is still in the preclinical stage of investigation. Therefore, understanding the mode and mechanism of action of Nrf2 due to these phytochemical substances is of great importance. Further research is needed to better understand the signaling mechanisms underlying the interaction between mitochondria and neuronal metabolism in order to develop therapies that can enhance antioxidant activity.

## 7. Conclusions

Many studies against oxidative stress point to the activation of the Nrf2 signaling pathway as the main mechanism of action related to the restoration of mitochondrial autophagy and anti-inflammatory mechanisms [117]. The importance of Nrf2 activators is not limited to their ability to counteract oxidative stress. This stress not only causes mitochondrial dysfunction but also a loss of control over the quality of the mitochondria themselves. This imbalance occurs when biogenesis is reduced and there is an accumulation of damaged mitochondria, which produce an excess of oxygen free radicals (ROS). Nrf2 positively influences mitochondrial biogenesis. Another mechanism involves interaction with p62/SQSTM1, which inactivates Keap1, allowing for Nrf2 translocation into the nucleus [44]. It is essential that Nrf2 activation maintains a dynamic balance, which is vital for mitochondrial stability. Although the Nrf2 system is positively linked to mitochondrial biogenesis and monitoring, as well as the control of their quality, the correlation between Nrf2 signaling and mitochondrial dynamics/mitophagy has not yet been thoroughly explored in the scientific literature. Currently, the nasal route of administration is at the center of research in the field of nanocarriers for the treatment of neurodegenerative diseases. Encapsulating the drug in the nanocarrier and administering it intranasally can improve its absorption and facilitate its transport. It has been found that liposomes encapsulating flavonoids improve the solubility and bioavailability of drugs and better penetrate the blood–brain barrier, counteracting oxidative stress by activating Nrf2 [120]. Generally, Nrf2 activation provides protection to cells and maintains their function. In contrast, prolonged Nrf2 activation appears to be detrimental, causing tissue damage, inflammation, and resistance to chemotherapy. Nrf2 inhibitors could be used to suppress prolonged Nrf2 activation due to defects in autophagy. The p62/SQSTM1-Nrf2 pathway has revealed the mechanistic basis of prolonged Nrf2 signaling. When autophagy is impaired, p62/SQSTM1 sequesters Keap1, causing the upregulation of Nrf2-controlled genes; this leads to prolonged Nrf2-ARE activation [39]. This review also opens the debate on the possibility that Nrf2 activation may enhance cannabinoid signaling through CB2R, amplifying the neuroprotective effects of ECBS on microglia reactivity and polarization. Although some details have yet to be clarified, these studies have provided new scenarios for the treatment of neurodegeneration. More insights into the mechanism of the interaction between the Nrf2-Keap1-ARE axis and autophagy are needed to facilitate the discovery of new therapies.

## Data Availability

Not applicable.
